# Developing anti-Müllerian hormone as an ovarian reserve biomarker

**DOI:** 10.1093/molehr/gaag021

**Published:** 2026-05-01

**Authors:** Bart C J M Fauser

**Affiliations:** University of Utrecht & University Medical Center Utrecht, Utrecht, The Netherlands

**Keywords:** anti-Müllerian hormone, ovary, follicle, polycystic ovary syndrome, premature ovarian insufficiency, IVF, pregnancy, fecundity

## Abstract

The first anti-Müllerian hormone (AMH) radioimmunoassay became available for clinical use in the early 2000s. This MHR Legacy Series review discusses key contributions from our own group towards establishing AMH as a robust ovarian reserve biomarker. Initial studies in female volunteers demonstrated decreasing serum AMH concentrations with increasing female age, along with a direct correlation between AMH levels and the number of antral follicles. The most relevant clinical conditions where measurements of serum AMH appear to be of great value include the diagnosis of various forms of ovarian dysfunction, especially polycystic ovary syndrome and premature ovarian insufficiency. In addition, a close correlation between initial serum AMH levels and ovarian response to standard stimulation for IVF has been firmly established, resulting in the development of a safe and effective AMH-based algorithm for individualized ovarian stimulation. Another area, beyond our own research, where AMH serum measurements have proven to be of great clinical significance is oncofertility. The capacity of AMH levels to predict fecundity—either spontaneous or following infertility treatment—or forecast age of menopause later in life remains uncertain and needs more well-designed, prospective, follow-up studies.

## Graphical abstract

**Graphical Abstract:**
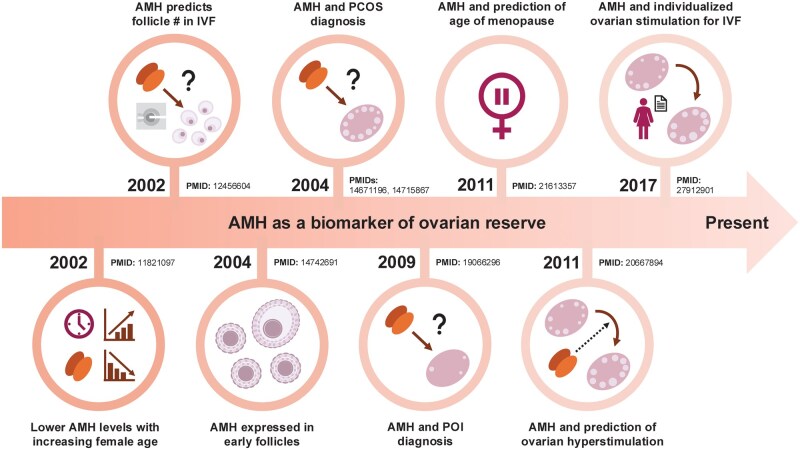
**Timeline of own contributions to developing anti-Müllerian hormone as a reliable, robust, and useful ovarian reserve biomarker.** AMH, anti-Müllerian hormone; PCOS, polycystic ovary syndrome; POI, premature ovarian insufficiency.

Anti-Müllerian hormone (AMH) is a secreted homodimeric glycoprotein, and a member of the transforming growth factor-β (TGF-β) superfamily. AMH is a non-covalently bound complex of a large N-terminal predomain and a smaller C-terminal signaling domain. AMH binds to the extracellular domains of the type-2 AMH receptor, followed by the release of the N-terminal domain that then binds to the type-1 activin receptor-like kinase (ALK)-receptor, resulting in the phosphorylation of intracellular SMAD (a term derived from its homology to the *C. elegans* SMA and *Drosophila* MAD families) and downstream protein signalling (for a review, see di Clemente *et al.*, 2021; Howard *et al.*, 2022) (Fig. 1).

**Figure 1.**
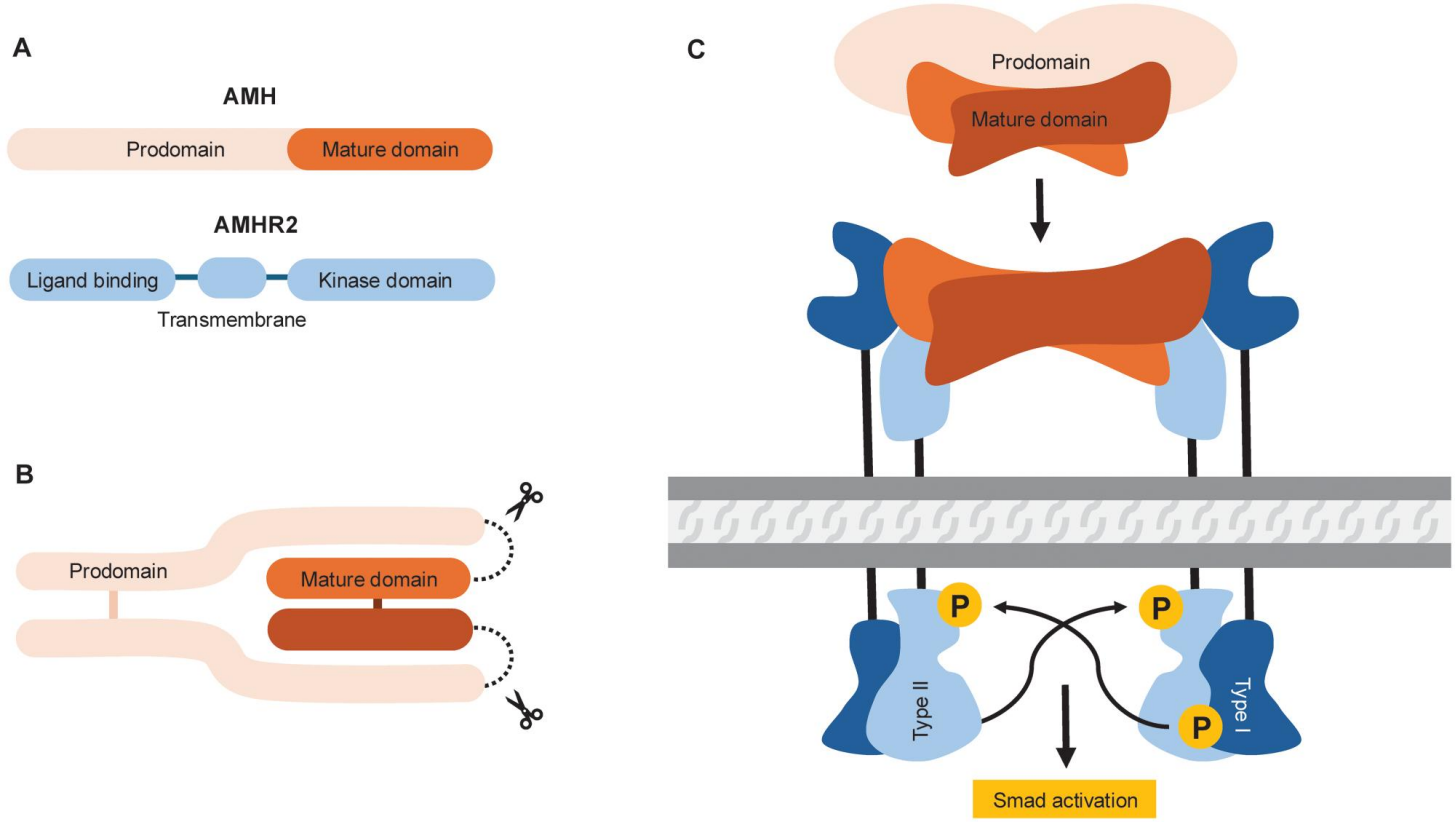
**Anti-Müllerian hormone receptor activation**. Schematic representation of (**A**) AMH and AMHR2, (**B**) assembly of AMH complex (with parts being cut), and (**C**) receptor binding and activation. Adapted from Howard *et al*. (2022) AMH, anti-Müllerian hormone; AMHR2, anti-Müllerian hormone receptor type 2.

AMH, first described by Alfred Jost in 1946, was initially referred to as Müllerian inhibiting substance (MIS), highlighting its role in male sexual differentiation during early human embryonic development by regulating regression of the Müllerian duct (Lee and Donahoe, 1993). Since the early 2000s, a distinct shift in attention regarding AMH research from the male towards the female has taken place. AMH has shed new light on a multitude of clinical conditions linked to female reproductive health and ovarian dysfunction.

Basic science colleagues (especially Prof. Themmen) working at the Department of Endocrinology & Reproduction at Erasmus University (Rotterdam, The Netherlands) provided groundbreaking novel insight concerning the potential crucial role of AMH in ovarian function. During their initial studies regarding the regulation of fertility in male and female mice, they were able to identify the AMH receptor (Baarends *et al.*, 1995). The observed expression patterns of the AMH receptor in ovarian follicles at different stages of development (primary and early secondary) suggested a role of AMH in the regulation of ovarian function. The AMH knock-out mice model allowed for a more detailed investigation of the presumed roles of AMH in early follicle development. Ovaries obtained from 25-day- and 4-month-old AMH knock-out mice contained more pre-antral and early antral follicles, whereas significantly less primordial follicles were observed in 4- and 13-month-old mice. This suggested crucial roles for AMH in regulating the initiation of growth of resting primordial follicles (Durlinger *et al.*, 1999) (Fig. 2). A collaborative project, funded by the European Union, between Professor Themmen from Rotterdam and Professor Groome from Oxford Brookes University, UK, led to the development of an AMH immunoassay that was sensitive enough to assess AMH serum concentrations in women (Al-Qahtani *et al.*, 2005). Furthermore, antibodies were developed that proved to be excellent tools to identify the types of follicles in mice and women that exhibit AMH expression.

**Figure 2.**
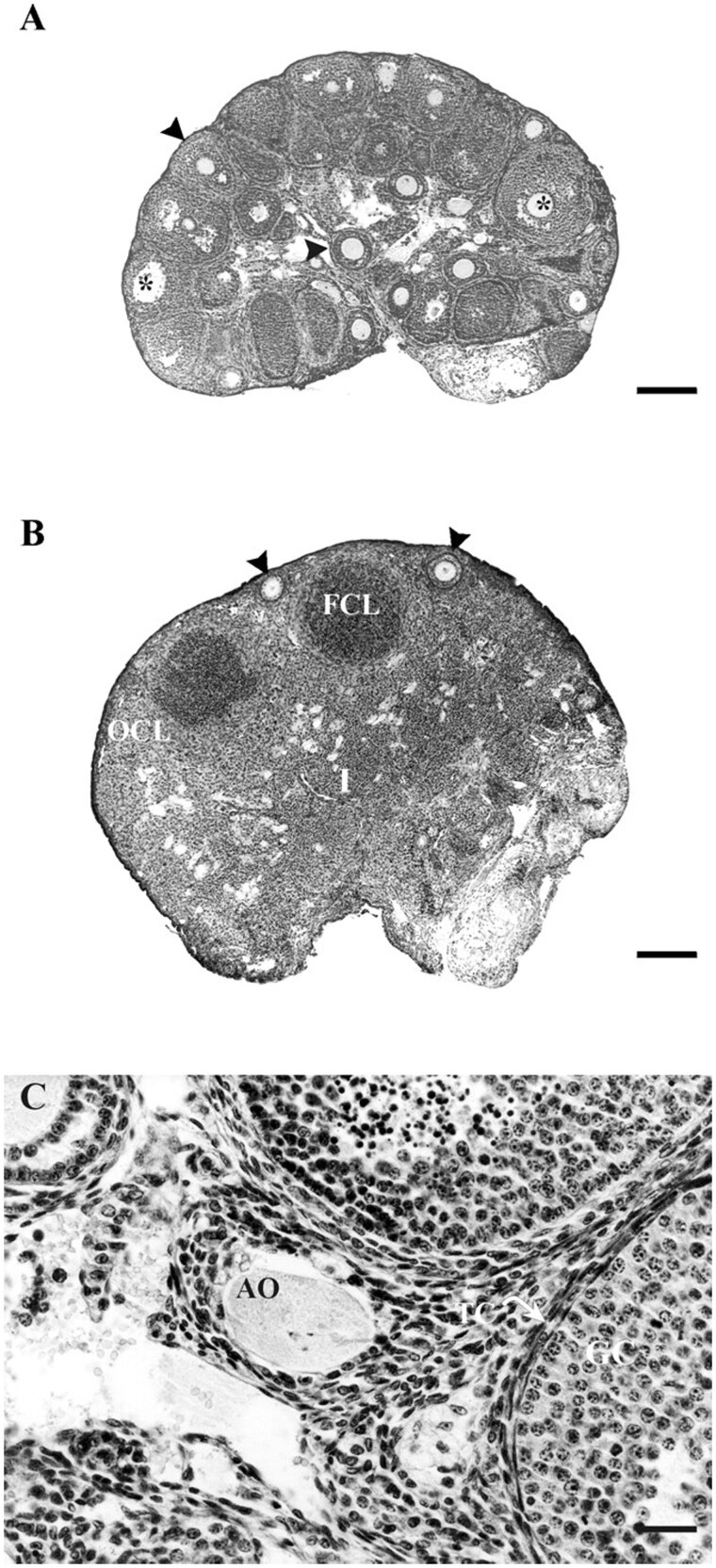
**Ovarian histology from anti-Müllerian hormone knock-out mice**. (**A**) Ovary of a 25-day-old mouse showing many non-atretic and atretic small developing follicles, whereas no corpora lutea can be detected. (**B**) Ovary of a 13-month-old mouse, mainly consisting of interstitial (I) and old (OCL) and fresh (FCL) corpora lutea, suggesting premature exhaustion. (**C**) Atretic oocyte (AO) from a 25-day-old knock-out mouse, with no clear granulosa cell and theca cell layer. Reproduced with permission from Durlinger *et al*. (1999).

Hence, AMH could potentially act as a crucial gatekeeper of follicle pool depletion and exhaustion. These novel findings concerning AMH in mice matched very well with my personal science history, having worked for several years in the basic science rat laboratory of Professor Aaron Hsueh (first at The University of California San Diego and later at Stanford University) aiming to identify novel intragonadal autocrine or paracrine factors regulating early follicle development and primordial follicle pool depletion (for a review, see Hsueh *et al.*, 2015).

This invited review—as part of the MHR Legacy Series—aims to highlight our early scientific contributions related to the potential roles of AMH in the elucidation of human ovarian function and dysfunction (for a review, see the graphical abstract and Fig. 3). This by no means implies that we were the only ones who contributed significantly to progress in this field, since—according to PubMed (searched 12 December 2025) using the keywords ‘AMH’, ‘ovary’, and ‘human’—2166 peer reviewed papers have been published since 2002 on the topic at hand.

**Figure 3.**
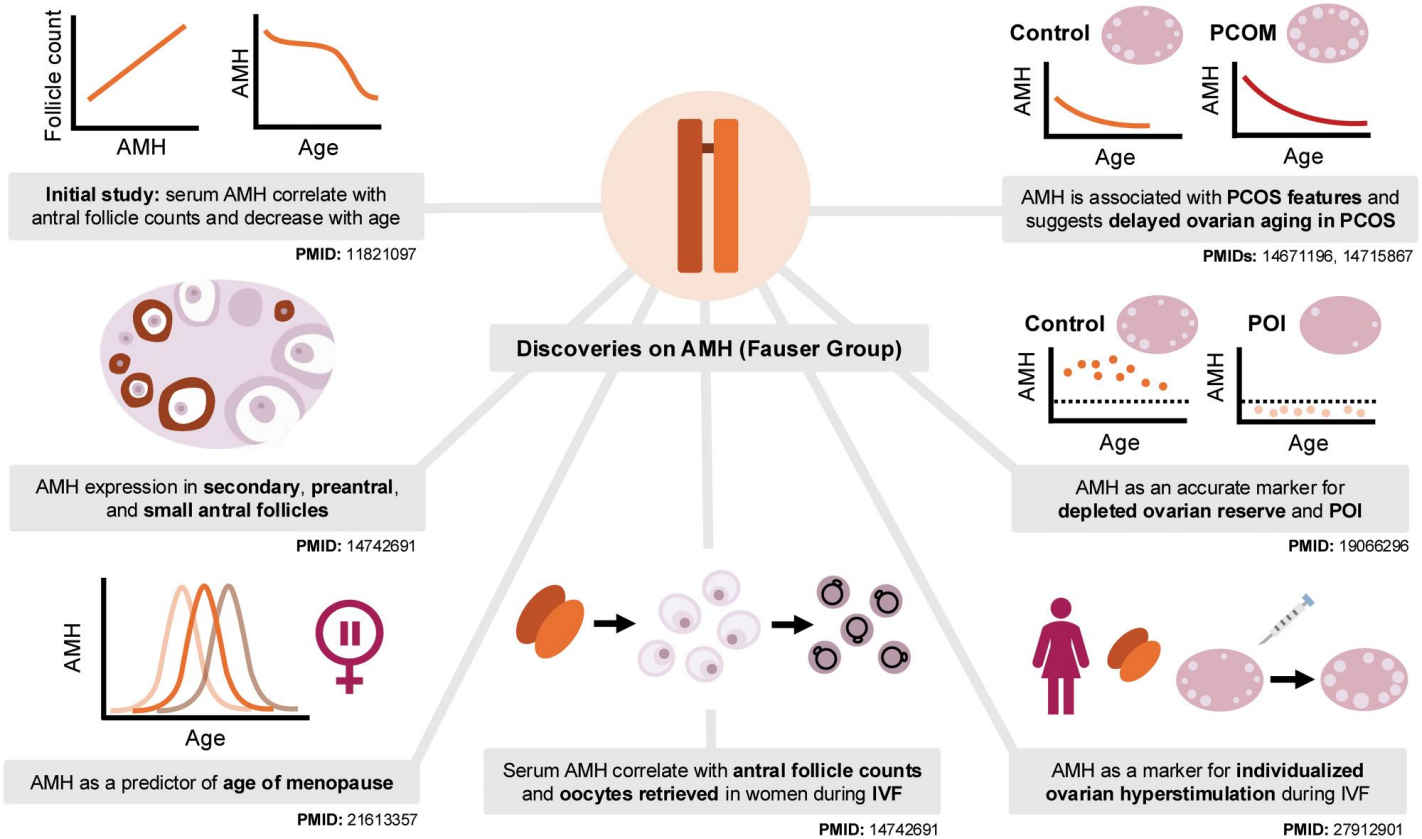
**More detailed representation of own studies concerning the potential role of anti-Müllerian hormone in female reproductive health**. AMH, anti-Müllerian hormone; PCOM, polycystic ovarian morphology; PCOS, polycystic ovary syndrome; POI, premature ovarian insufficiency.

## Initial studies established AMH as a robust ovarian reserve biomarker

**Figure 4.**
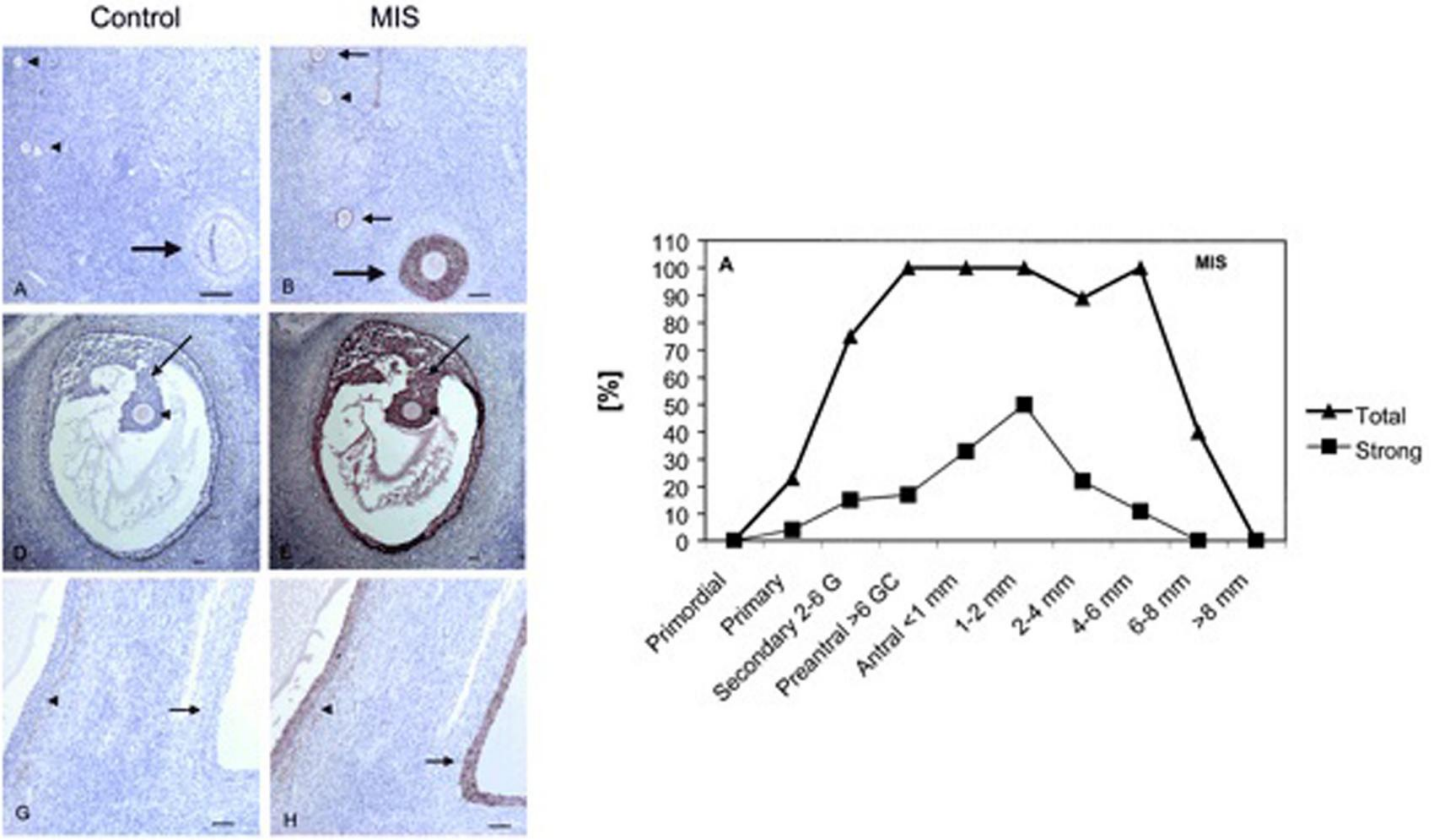
**Anti-Müllerian hormone immunohistochemically stained human ovarian tissues**. Left: (**A, D, G**) controls, (**B, E, H**) stained using AMH antibody. (A, B) section at 100× magnification with primordial follicles. (D, E) adjacent section (40×) with small antral follicles. (G, H) adjacent section (100×) with two large antral follicles. Right: Graphical depiction of the percentage of follicles with strong and total AMH staining in relation to stages of follicle development. Staining increased rapidly with the stage of follicle development and decreased beyond 4–6 mm diameter. Reproduced with permission from Weenen *et al*. (2004). AMH, anti-Müllerian hormone; GC, granulosa cell; MIS, Müllerian inhibiting substance (AMH).

Our initial preliminary study, which took place in 41 young healthy normo-ovulatory volunteers, demonstrated for the first time that serum AMH concentrations decrease with increasing female age, and that AMH levels correlate well with the number of antral follicles (antral follicle count; AFC), as assessed by transvaginal ultrasound scanning (de Vet *et al.*, 2002) (Fig. 3). More recently, this work was recognized as one of the most influential papers so far published in *Fertility and Sterility* (Niederberger *et al.*, 2018). Using immunohistochemistry, we also investigated AMH expression patterns in human ovarian tissue sections obtained from 12 healthy, regularly cycling women who underwent surgery for benign gynecological reasons. This showed high AMH staining in the granulosa cells of secondary, preantral, and small antral follicles, along with absent AMH expression in primordial and large pre-ovulatory follicles. These preliminary findings were published in *Molecular Human Reproduction* (Weenen *et al.*, 2004) (Fig. 4). Such initial observations underline a critical role of AMH in early human follicle development, especially the recruitment of resting primordial follicles. Later on, others provided additional pivotal evidence that confirmed serum AMH levels indeed directly correlate with the size of the ovarian primordial follicle pool—in human ovarian tissue obtained from 42 healthy women between 26 and 52 years of age undergoing oophorectomy for benign gynecological reasons (Hansen *et al.*, 2011).

Over subsequent years, evidence rapidly accumulated that serum AMH represents the best available biomarker for diminished ovarian reserve—associated with reduced female reproductive potential—as compared to chronological age, AFC, or FSH levels. However, various endpoints used in initial studies concerning AMH and ovarian reserve all represent surrogate markers of diminished ovarian reserve. Ovarian follicle pool depletion eventually ends in exhaustion of the stock of primordial follicles, resulting in menopause, representing the end of female reproductive life. Hence, the key question remained whether serum AMH levels assessed at a younger age indeed correlate over time with the real-life endpoint—i.e. the age of menopause. Our initial study addressing this vital question involved an 11-year follow-up of a cohort of 257 normo-ovulatory women between 21 and 46 years of age (Broer *et al.*, 2011a). Nineteen percent of women in the initial cohort had reached postmenopause, and age, AFC, and AMH were all significantly correlated with the age of menopause. Numerous well-designed studies have followed since then (for a review, see Nelson *et al.*, 2023). The robust assessment of individual variation in ovarian aging (for a review, see Broekmans *et al.*, 2009) and its associated varying extent of diminished fertility with increasing female age still represents the most crucial challenge in fertility care today.

## AMH as a marker of ovarian function and dysfunction

Another study, undertaken by us in the early years, which involved 119 patients undergoing IVF, demonstrated that serum AMH concentrations upon initial screening were highly correlated with AFC. More importantly, initial AMH levels were associated with the number of oocytes retrieved for IVF following standard ovarian stimulation (van Rooij *et al.*, 2002) (Fig. 3). Numerous subsequent studies convincingly demonstrated that serum AMH represents the best baseline biomarker capable of predicting which women exhibit increased chances to either hyperrespond (which means increased chances for treatment complications such as ovarian hyperstimulation syndrome, which may require cancellation of the IVF treatment) or to hyporespond (which means poor IVF success chances due to a low number of oocytes) to ovarian stimulation (Broer *et al.*, 2011b).

Another initial study, involving 128 women diagnosed with normogonadotrophic (WHO class 2) anovulatory infertility, demonstrated for the first time that: (i) serum AMH levels were distinctly elevated as compared with regularly cycling age-matched controls, (ii) serum AMH levels were clearly associated with polycystic ovary syndrome (PCOS) features, and (iii) serum AMH levels showed a less pronounced decrease over time, suggesting retarded ovarian aging in women with PCOS (Laven *et al.*, 2004; Mulders *et al.*, 2004) (Fig. 3).

In a nationwide study involving 10 Dutch hospitals and a total of 342 women, we were able to demonstrate that all women previously diagnosed with premature ovarian insufficiency (POI) presented with serum AMH levels below the fifth percentile of age-matched, normo-ovulatory women (Knauff *et al.*, 2009) (Fig. 3). More importantly, additional analyses revealed that serum AMH was more consistently correlated with early signs of follicle pool depletion in women that did not (yet) fulfill all criteria for POI diagnosis such as elevated FSH levels (above 25 or 40 IU/ml) and amenorrhea.

We were also able to demonstrate an important role for serum AMH levels in predicting the recovery of ovarian function in 61 young, hypogonadotropic anovulatory women diagnosed with anorexia nervosa during weight gain interventions (van Elburg *et al.*, 2007). Next to weight gain itself, initial ovarian markers like FSH, inhibin B, and AMH are able to predict resumption of menses using multivariate analysis with time to recovery as the main outcome measure.

Throughout a woman’s life, the stock of resting primordial follicles diminishes, as stated earlier. We could only imagine a single condition where the primordial follicle quantity would actually increase, i.e. following the auto-transplantation of cryopreserved ovarian tissue. Since the early 2000s, women of reproductive age who require gonadotoxic cancer treatment have been given the opportunity for their ovarian tissue to be cryopreserved with the purpose of allowing them to fulfill their reproductive desires once cured of cancer. Hundreds of children have already been born following ovarian tissue auto-transplantation. However, the extent and duration of recovery of ovarian function vary significantly from one person to the next. We anticipated that the assessment of serum AMH concentrations following such a procedure would help to better predict a patient’s individual chances for recovery of ovarian function, as well as its duration until complete exhaustion. To our great surprise, and for unknown reasons, this was not the case in a preliminary study that we undertook in 10 women during a 2.5-year follow-up in collaboration with Professor Donnez from Brussels (Janse *et al.*, 2011). It is possible that changes in ovarian vascularization prevent serum AMH levels from representing early follicle development within the ovaries.

## AMH: current use and overuse

In discussions concerning the clinical implications of AMH serum assays, the most crucial component is probably ongoing concerns about the quality of the assay itself. The following issues should be considered: (i) The use of different assays with different sensitivity and specificity also generates different absolute concentrations, which renders the direct comparison of different assays problematic. (ii) The lack of internationally accepted standardization and calibration. (iii) Potential interfering effects of long-term sample storage, body mass index, the vitamin status, and ethnicity. (iv) The existence of AMH isoforms (resulting from cleavage of the secreted precursor hormone) with a varying degree of biological activity further complicates AMH measurement (Punchoo and Bhoora, 2021).

The capability of initial serum AMH levels as a predictor of ovarian response to stimulation for IVF has been clearly established by our group using individual patient data (IPD) meta-analyses involving a multitude of studies and thousands of women (Broer *et al.*, 2011b). As it has been convincingly demonstrated that AMH is associated with ovarian response, the concept has subsequently been tested to determine whether initial AMH levels (along with body weight) could be incorporated in a first ‘companion diagnostic’ approach for ovarian stimulation in fertility care. Hence, could an algorithm be developed to inform individualized dosing of a newly developed human recombinant FSH preparation? A safe and effective IVF ovarian stimulation regimen was considered to generate between 8 and 14 oocytes upon retrieval. Indeed, a first large sample size multi-center randomized control trial (RCT) was able to demonstrate a higher proportion of patients reaching the desired oocyte range upon retrieval as compared to standard dosing (Nyboe Andersen *et al.*, 2017). The validity and robustness of the algorithm have subsequently been confirmed by multiple additional RCTs, along with real-world evidence from various regions of the world. Patient-tailored, algorithm-based ovarian stimulation regimens result in safer and more efficient stimulation protocols for IVF. Unfortunately, AMH levels alone cannot be used to predict individual IVF success chances.

Another area where AMH has proven to be of great clinical benefit is for the monitoring of ovarian function in women with cancer who are undergoing gonadotoxic treatment (for a review, see Anderson *et al.*, 2022). A measured serum AMH level can be used to predict the extent of ovarian damage following various chemotherapy or radiation regimens and identify individuals who are particularly prone to irreversible ovarian damage due to chemotherapy, as well as individual chances for the recovery or irreversible loss of ovarian function following the completion of treatment.

Although serum AMH levels have been recommended in the updated 2023 ‘evidence-based’ PCOS guideline (Teede *et al.*, 2023) as an appropriate substitute for ultrasound diagnosis of polycystic ovaries, a recent systematic review and meta-analysis, involving a total of 68 studies, was unable to determine suitable AMH cut-off levels for PCOS diagnosis (van der Ham *et al.*, 2024).

Unfortunately, little effort has been undertaken since our initial observations to further elucidate a role for AMH in early POI diagnosis. Even in the recently updated POI guideline, AMH measurements are not recommended in the context of POI diagnosis (Panay *et al.*, 2025).

Despite much ongoing speculation regarding a possible discrepancy between oocyte quantity and quality, I remain unconvinced that this is indeed the case. Unsurprisingly, measured AMH levels are not able to differentiate between oocyte quality independent from follicle quantity (Lie Fong *et al.*, 2008).

The potential value of measured AMH levels as a predictive tool is also being increasingly explored in infertile women with other disease conditions, such as endometriosis or uterine fibroids, with or without particular ovarian involvement or surgery (Valenti *et al.*, 2026).

A recent study has assessed the ability of measured AMH levels to predict ‘spontaneous’ pregnancy chances (after intercourse without medical intervention), or time to pregnancy in young women who try to conceive after stopping contraception (Nelson *et al.*, 2024). If this were indeed the case, such a test could assist young women in making more personalized reproductive choices, especially when AMH can be reliably assessed during steroid contraception use. Such tests are already widely offered over the counter by current e-health and FemTech companies worldwide. However, offering such tests is clearly premature and should not be currently recommended due to misleading claims (Johnson *et al.*, 2023) and insufficient clinical evidence (Copp *et al.*, 2024). Beyond doubt, many confounders could be interfering with a direct correlation between AMH concentrations and fecundity, either without medical intervention, or following medical treatment of anovulatory infertility (i.e. ovulation induction) or IVF. Such factors may include sperm quality, frequency and timing of intercourse, emotional stress, environmental factors, obesity, or concomitant disease.

## AMH: key future challenges

**Figure 5.**
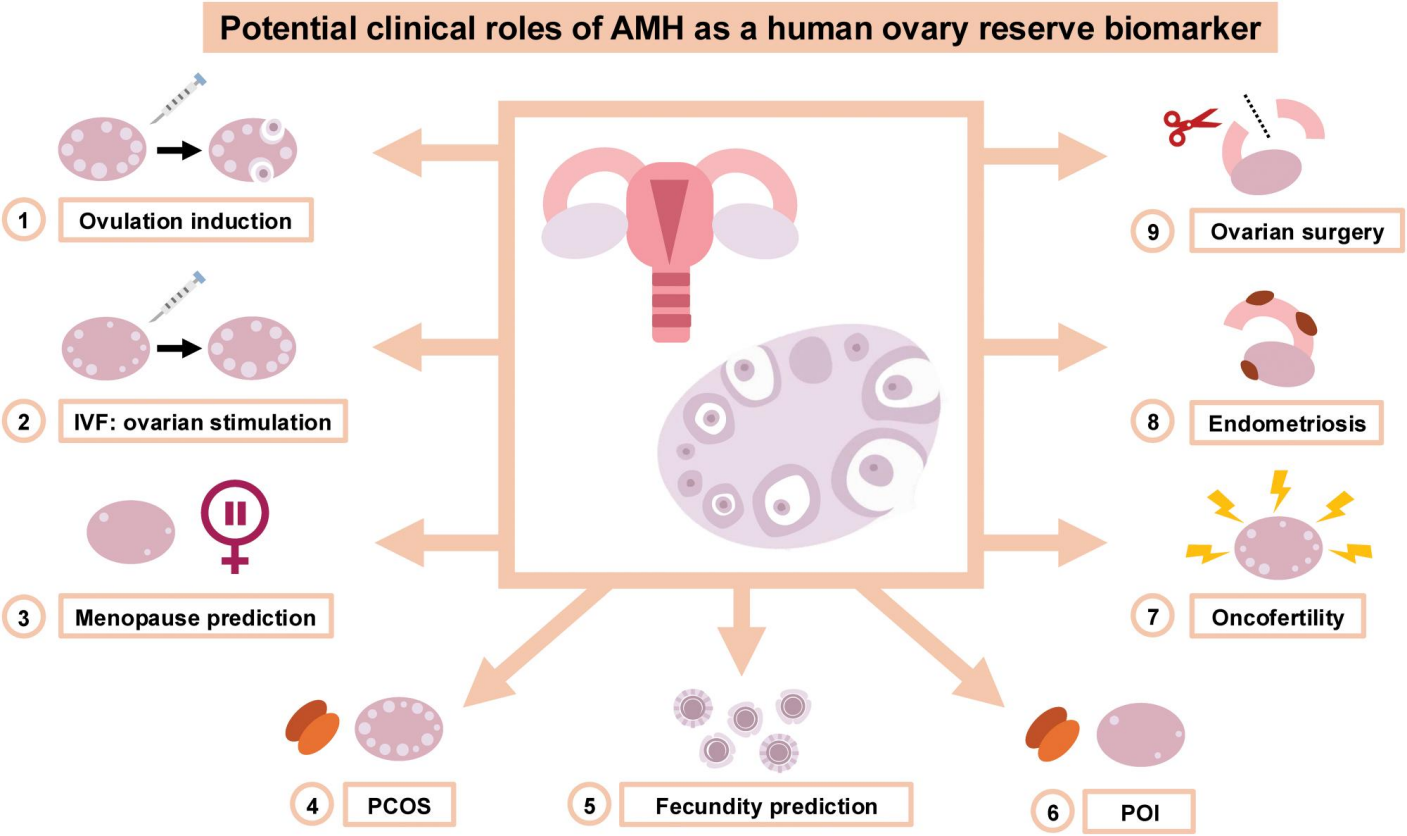
**Summary representation of the potential clinical roles of anti-Müllerian hormone as a human ovarian reserve biomarker**. PCOS, polycystic ovary syndrome; POI, premature ovarian insufficiency.

In conclusion, the possibility of measuring AMH serum concentrations, which arose in the beginning of this millennium, has allowed for a true breakthrough in female reproductive health assessment (Fig. 5). AMH has been demonstrated to be the most robust currently available biomarker for ovarian reserve. Its potential clinical implications have been demonstrated in assessing ovarian function and dysfunction, especially related to fertility and infertility treatment (Broer *et al.*, 2014). Beyond doubt, the AMH story is still a ‘work in progress’ and deserves continued high-quality investigation. Most importantly, more robust AMH assays with improved standardization and less variation (Fauser and Nelson, 2020) will be key to further progression in understanding human ovarian function and dysfunction.

The most promising areas include:

- With advances in developing bioengineering tools for the study of female reproduction (for a review, see Francés-Herrero *et al.*, 2022), three-dimensional artificial ovaries may be generated which could prove a useful tool for the more detailed study of the role of AMH in regulating primordial follicle arrest or growth, its potential interaction with other factors, and the presumed inhibitory roles of AMH in later stages of gonadotropin-dependent follicle development.
- AMH levels as an additional tool for (early) diagnosis and follow-up over time regarding the type and extent of the disease for women presenting with various forms of ovarian dysfunction (especially PCOS and POI).
- AMH levels as an additional tool to fine-tune safe and effective patient-tailored ovarian stimulation for various forms of infertility treatment.
- AMH levels should be explored in greater detail as a potential predictor of pregnancy chances without medical intervention, and the chances of success of various infertility treatments such as ovulation induction, IVF, endometriosis, or ovarian surgery.
- A role for AMH measurements in oncofertility should be further advanced. AMH levels may help to identify women at risk for irreversible loss of ovarian function due to gonadotoxic cancer treatment, to identify the least damaging cancer treatment, to follow recovery of ovarian function after cessation of treatment, and predict future fertility potential.
- More information is needed regarding the capacity of AMH levels, assessed at a young age, to reliably predict the age of menopause. It is possible that the combined use of measured serum AMH concentrations along with contemporary genomic data related to the age of menopause may significantly improve the predictive potential. More individualized knowledge regarding the age of menopause may be crucial not only in relation to infertility that precedes menopause (hence, early menopause means a decrease in natural fertility at an earlier age), but also for long-term quality of life and cardiometabolic health.
- The preclinical development of both AMH agonists and antagonists is still ongoing, and unfortunately, these are not yet ready for studies in humans. The availability of such compounds could, in the future, allow the modification of the age of menopause, with distinct implications for both the fertility decrease preceding menopause and later-life health. The ability to extend female reproductive life would represent a true revolution.

## Author Biography

**Bart C.J.M. Fauser, MD, PhD, FRCOG**, is a Professor Emeritus of Reproductive Medicine, Gynecologist, former Head of the Department for Reproductive Medicine and Gynecology, and former Chair of the Division of Woman & Baby at the University of Utrecht and the University Medical Center Utrecht, The Netherlands.

He currently acts as the scientific director of the International Federation of Fertility Societies (IFFS), an international member of the US National Academy of Medicine (NAM), member of the Academia Europea, Honorary member of ESHRE, Fellow ad Eundum of the Royal College of Obstetrics and Gynaecology (RCOG), consultant and advisory board member. He is a past Editor-in-Chief of *Human Reproduction Update* and, more recently, *Reproductive Biomedicine Online* (RBMO).

He previously held posts as a Fulbright post-doctoral scholar at the University of California, San Diego, CA, USA, visiting professorships at Stanford University School of Medicine (CA, USA), Free University of Brussels (Belgium), University of Siena (Italy), University of Southampton (UK), the University of Adelaide (Australia), and full professorship at the Erasmus University, Rotterdam, The Netherlands.

Professor Fauser’s primary research interest has been ovarian function and dysfunction, and he is amongst the most cited authors in Reproductive Medicine worldwide, with over 500 peer-reviewed scientific publications and a Hirsch factor of over 120 (Scopus).
